# The Potential for Reusing Superabsorbent Polymer from Baby Diapers for Water Retention in Agriculture

**DOI:** 10.3390/gels11100795

**Published:** 2025-10-02

**Authors:** Kamilla B. Shishkhanova, Vyacheslav S. Molchanov, Ilya V. Prokopiv, Alexei R. Khokhlov, Olga E. Philippova

**Affiliations:** 1 A.N. Nesmeyanov Institute of Organoelement Compounds, Russian Academy of Sciences, 119991 Moscow, Russia; shishkhanova@polly.phys.msu.ru (K.B.S.); prokopiv@polly.phys.msu.ru (I.V.P.); khokhlov@polly.phys.msu.ru (A.R.K.); 2 Physics Department, Lomonosov Moscow State University, 119991 Moscow, Russia; 3 Institute of Chemistry, Saint Petersburg State University, 199034 Saint Petersburg, Russia

**Keywords:** hydrogels, cross-linked polymers, superabsorbent polymers, swelling, water retention capacity

## Abstract

Annually, about 2.4 million tons of superabsorbent polymers (SAPs) used in disposable diapers are thrown away, polluting our planet. This study aims to explore the potential for reusing SAPs removed from diapers to enhance soil water retention. To this end, the swelling and water retention properties of SAP gels from three different types of diapers were compared to those of an agricultural gel, Aquasorb. Sand was used as a model for soil. When mixed with sand, diaper gels have a swelling degree of ca. 100 g per gram of dried polymer, and a swelling pressure of 12–26 kPa, which are similar to those of Aquasorb gel. Using a synthesized poly(acrylamide-co-sodium acrylate) gel as an example, the correlation between the swelling pressure and the compression modulus of the swollen gel was demonstrated. Soil-hydrological constants were estimated from water retention curves obtained by equilibrium centrifugation of gel/sand mixtures. It was observed that adding 0.3 vol% of diaper gels to sand leads to a 3–4-fold increase in water range available to plants, which is close to that provided by agricultural gel Aquasorb. The water-holding properties were shown to be maintained during several swelling/deswelling cycles in the sand medium. The addition of diaper gels to soil had a significant positive impact on mustard (*Brassica juncea* L.) seed germination and seedling growth, similar to the agricultural gel Aquasorb. This suggests high potential for the reuse of SAPs from diaper waste to improve soil water retention and water accessibility to plants. This would provide both economic and environmental benefits, conserving energy and raw materials to produce new agricultural gels and limiting the amount of waste.

## 1. Introduction

The increasing production of synthetic polymers, especially those that are disposed after single use, presents many ecological challenges. In particular, it concerns disposable baby diapers. The current practice of discarding diapers results in the loss of valuable materials, such as superabsorbent polymers (SAPs), which account for approximately one third of the diaper’s weight [1], and the generation of large amounts of municipal solid waste [2]. By 2022–2023, the global annual production of SAPs had reached 3.5 million tons, and it is expected to increase to 5.3 million tons by 2030 [3]. According to Global Market Insights [4], the baby diaper segment accounts for 69.8% of the SAP market. This means that about 2.4 million tons of these polymers are used in the production of diapers, and the same amount is discarded after use, polluting our planet.

The problem can be partially solved by reusing SAPs from diapers. Analysis carried out by Takaya et al. [5] has shown that SAP is one of the most readily recoverable components from diapers, and it can find use in sectors such as construction and agricultural industries.

We believe that agriculture is the most promising area for the reuse of SAPs from diapers. Most often, diaper SAPs represent slightly crosslinked sodium polyacrylate gels [5,6]. Similar gels based on copolymers of potassium acrylate and acrylamide [7,8] are already used in agriculture for various purposes, including water retention, soil management, and fertilizer delivery, thereby supporting crop production and helping to address water scarcity issues.

The most important function of SAP gels is water retention. The lack of water and the desertification of soil are among the most serious anthropogenic problems affecting approximately one third of the land surface of the world [9]. SAP gels can absorb water in quantities several hundred times higher than their dry weight [10]. When SAP is incorporated into the soil, it absorbs rain or irrigation water and acts as a reservoir, slowly releasing water to the roots through osmotic pressure differences, thus providing a continuous water supply for plants and reducing water loss through drainage and evaporation [11,12,13,14,15,16,17,18]. The use of gels reduces water consumption for irrigation and permits significantly increased crop yield while using less water [19]. For example, Zhu et al. [14] demonstrated on the example of the common Asian leafy vegetable choi sum (also known as Chinese cauliflower) that SAP gel can increase the irrigation water use efficiency by more than 60%. By using hydrogel, the frequency of watering could be reduced from 21 times per week to 7 times, resulting in a saving of 196,000 L of water per crop cycle, based on a per-hectare estimation.

In soil management, SAPs are used as soil conditioners. Their expansion and shrinking during the water cycling improve the permeability, texture, and aeration of the soil [13,20,21]. Also, SAPs can enhance the soil’s resistance to erosion due to the formation of a protective layer on the soil surface, consisting of dried SAP film and soil particles [15].

In fertilizer delivery, SAP gels are used as slow- and controlled-release carriers. They can prevent fertilizer loss by leaching from the root zone into groundwater or by volatilization, as well as reduce the application frequency of fertilizers and the potential negative effects of overdosage [20,21,22,23,24,25,26,27,28,29]. Plant protection products, such as insecticides or herbicides, can also be loaded onto the gels to prevent unnecessary contamination and minimize environmental impact.

First attempts to demonstrate the technical feasibility of using SAP from waste baby diapers to increase water content in soil were carried out recently by Sánchez-Orozco et al. [30] and Al-Jabari et al. [31]. They studied the free swelling and deswelling of recovered SAPs as well as the water accumulation by SAP/soil mixtures. It was shown that sandy [30] and red [31] soils mixed with SAP can lose water more slowly than untreated soil. For instance, after 12 days, the loss of water in the SAP/red soil sample was 76%, compared to 80.6% for the control soil sample without SAP [31]. Also, the addition of recovered SAP in soil has been shown to enhance seedling growth of bean (*Phaseolus vulgaris* L.) and pumpkin (*C. pepo*) [30] and to favor tomato growth [31]. In further studies [32], the water holding capacity of soil with recovered SAP was investigated during several wetting and drying cycles. It was found that, after five cycles, the water holding capacity decreased by 5% at a SAP concentration of 1.6 wt% [32].

At the same time, in these experiments, the hydrophysical characteristics of soil loaded with diaper gels, including the amount of water available for plants, were not determined. Additionally, in real agricultural conditions, the gels are forced to swell in a limited pore space under the pressure of soil particles, and their behavior is determined by the balance between the elasticity of the swollen gel and the resistance of the soil particles [33]. In this case, in addition to water retention, it is also important to determine the gel swelling pressure characterizing the elasticity of the swollen gel. However, no data is currently available on the swelling pressure or even on the degree of swelling of diaper gels mixed with soil.

The purpose of this study is to compare the water retention capacity of SAP gels from different types of disposable diapers with that of a typical agricultural gel, Aquasorb. Sand is used as a model for soil. Our main contribution lies in the determination of the hydrophysical properties of sand loaded with the gels, including the amount of water available for plants, and the swelling pressure, which characterizes the mechanical properties of swollen gels confined in the sand. We also aim to establish a correlation between the swelling pressure and compression modulus of hydrogels. The research clearly shows that the water retention capacity of SAP gels removed from diapers and mixed with sand is similar to that of the agricultural gel, opening up the possibility for their widespread use in agriculture.

## 2. Results and Discussion

### 2.1. Characterization of Gel Samples

**Figure 1:**
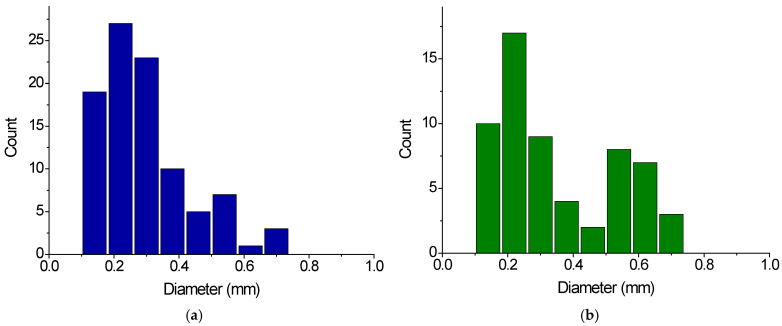

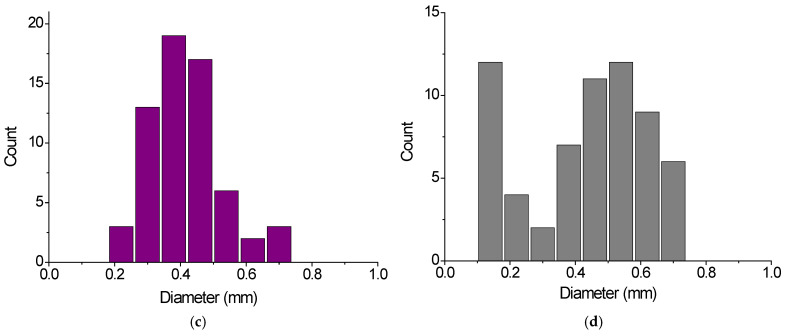
Histograms of dry gel particle size distribution for diaper gels Huggies (**a**), GooN (**b**), Merries (**c**), and agricultural gel Aquasorb (**d**).

For the study, we chose three samples of SAP gels removed from diapers Huggies, GooN, and Merries. The size of the removed gel particles was determined by optical microscopy. The histograms of size distribution are presented in Figure 1a–c. The size of gel particles in all samples falls into the range of 0.1–0.75 mm. The distributions are monomodal for Huggies and Merries gels with maxima at 0.2–0.25 and 0.4 mm, respectively, and bimodal for GooN gel with two maxima at 0.2–0.25 and 0.55–0.6 mm (Table 1).

**Figure 2:**
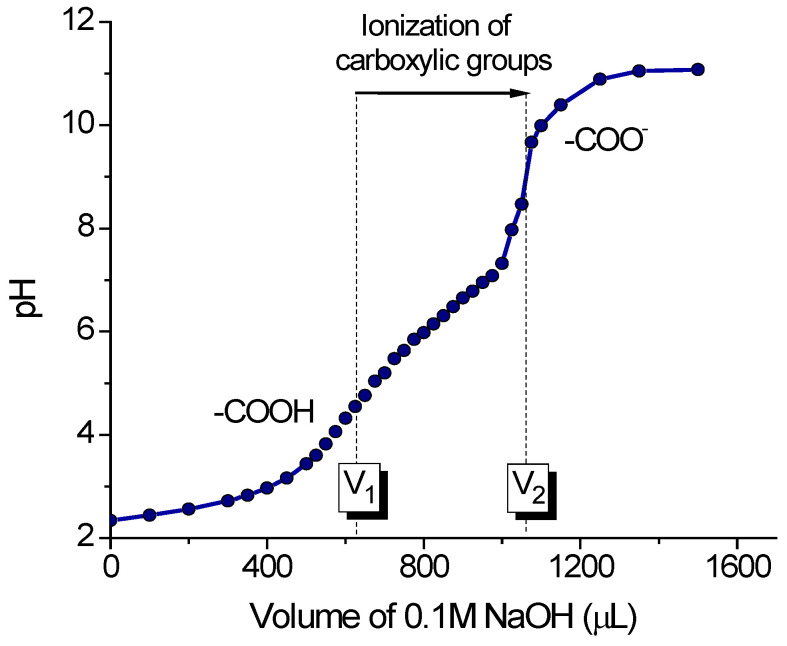
Potentiometric titration curve of 10 mL of 0.12 wt.% aqueous suspension of Huggies gel particles by 0.1 M NaOH. First inflection point V_1_ corresponds to the titration of HCl excess, and second inflection point V_2_ corresponds to the titration of gel carboxylic groups.

The diaper gels are typically composed of sodium polyacrylate crosslinked by poly(ethylene glycol) diacrylate [6]. The content of charged COO^-^ groups was determined by potentiometric titration, after converting the carboxylic groups to the protonated form by adding 1 M HCl. A typical titration curve is presented in Figure 2. Similar curves were obtained for all diaper gel samples. The curve has two distinct inflection points. The first one corresponds to the titration of HCl excess (V_1_), the second one corresponds to the titration of the gel carboxylic groups (V_2_) [34]. From the difference in the titrant volumes (V_2_ − V_1_), the content of carboxylic groups (in mmoles per 1 g of dried gel) was estimated. The results are presented in Table 1.

Aquasorb was used as an example of an agricultural gel. It is supplied by the manufacturer as a dry granular material. A histogram of the size distribution of grains, determined by optical microscopy, is presented in Figure 1d. It is bimodal. The average grain size is given in Table 1. It is close to the size of gel particles used in diapers (Table 1). Aquasorb gel is composed of a copolymer of potassium acrylate and acrylamide crosslinked by N,N′-methylenebisacrylamide [6,7]. According to potentiometric titration data, it contains 1.8 mmoles of COO^-^ per gram of dry gel (Table 1).

Thus, the size of diaper gel particles is close to the size of agricultural gel particles, but their degree of charging determined by the content of charged COO^-^ groups is two-fold higher.

### 2.2. Water Retention Capacity

#### 2.2.1. Swelling

Water retention is one of the main characteristics of SAP gels used in agriculture. It depends primarily on the swelling ability of the gels, which is characterized by the degree of swelling Q determined as [35]:

(1) $$Q=\frac{m_{sw}-m_{dry}}{m_{dry}}\;\;$$

where $m_{sw}$ and $m_{dry}\;$are the weights of swollen and dry gel, respectively.

**Figure 3:**
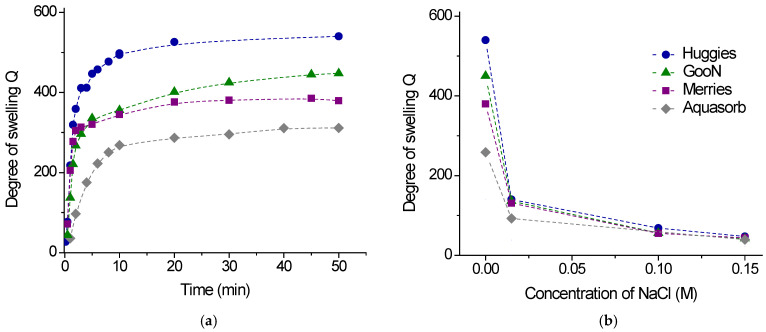
(**a**) Kinetics of free swelling in water of diaper gels Huggies (circles), GooN (triangles), Merries (squares), and agricultural gel Aquasorb (diamonds). (**b**) Effect of NaCl concentration on the free swelling of the same gels in water.

Figure 3a shows the kinetics of the swelling in water for the gels taken from three samples of diapers (Huggies, GooN and Merries). One can see that upon immersion of the dry gel samples in a large volume of water the gels swell fast, acquiring the equilibrium swelling in only 3–5 min. The resulting degrees of swelling Q are very high and reach 540 g of water per 1 g of dry gel (Table 2). This means that the gel absorbs water 540 times its own weight. Most obviously, such high swelling is due to the presence of charged groups on the network chains and their counterions. Similarly charged groups on the network chains induce electrostatic repulsion within the gel, whereas the counterions induce exerting osmotic pressure. Both factors promote gel swelling. When salt is added to water, the degree of swelling decreases, reaching 42–47 in 0.15 M NaCl (Figure 3b). This is a common behavior of polyelectrolyte gels [30,31,36]. It is due to the screening of electrostatic repulsion by salt, as well as the decrease in the osmotic pressure of counterions. These results suggest that the performance of SAP may vary depending on the irrigation water, which contains different ions, and the salinity of the soil.

Figure 3 allows for comparison of the data for free swelling for the diaper gels and the gel for agricultural use, Aquasorb. One can see that the diaper gels swell much faster than the agricultural gel, and their equilibrium degree of swelling Q is higher (Table 2). This may be due to the higher content of charged units and/or the lower cross-linking density in diaper gels. In 0.15 M NaCl, the degree of swelling for Aquasorb is still smaller than for diaper gels (Figure 3b). Thus, free swelling is more significant in diaper gels compared to the agricultural gel.

But, for the agricultural needs, it is important to consider the swelling of the gel confined in a porous medium, rather than the free swelling. This is because the gel should swell and retain water within the soil pores. For this reason, we also investigated the swelling of gel particles mixed with sand. The content of dried SAP gels in the sand was 0.3 vol%, as recommended by the manufacturer of Aquasorb. The experiments were performed in a container closed by a piston to maintain a constant volume of the system. This models the swelling of a gel that is deeply immersed in soil.

The degree of swelling of gel particles mixed with sand $Q_{mix}$ was determined as [15]:

(2) $$Q_{mix}=\frac{{m_{sw\_mix}-m}_{sw\_sand}\;{-\;m}_{dry}}{m_{dry}}$$

where $m_{sw\_mix}$ and $m_{sw\_sand}\;$are the weights of swollen gel/sand mixture and swollen sand, respectively. From Table 2, it is seen that compared to free swelling, the swelling in sand pores is much smaller. Similar behavior has been previously observed for various gels [7,15,19,34,37,38]. It was attributed to restricted volume available for swelling and to the mechanical resistance of the sand particles [34]. Table 2 shows that the decrease in swelling is much more significant for diaper gels (by a factor of 4.1 to 5.5) than for Aquasorb (by a factor of 3.1). Finally, in a porous medium, the swelling of diaper gels becomes close to that of Aquasorb. Thus, in all gels mixed with sand, the degree of swelling remains fairly high (ca. 100), indicating that both types of gels are suitable for water retention.

#### 2.2.2. Water Retention

**Figure 4:**
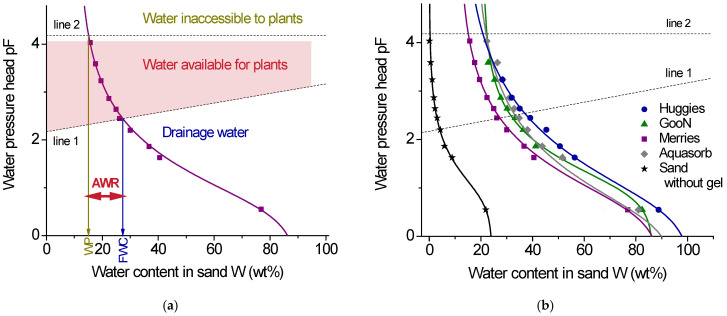
(**a**) Illustration of the estimation of the soil-hydrological constants FWC and WP on an example of water retention curve for sand with 0.3 vol% of Merries gel. pF = log(−P), where P is water head pressure in cm. Dotted lines represent straight lines that correspond to the following equations: pF = 2.17 + W/100 (line 1) and pF = 4.18 (line 2), which limit the zones of drainage water, water available to plants, and water inaccessible to plants. The points of intersection of lines 1 and 2 with the water retention curve are used to estimate the soil-hydrological constants FWC and WP, respectively [40]. From the difference between FWC and WP values, the range of water available to plants, AWR is determined. (**b**) Water retention curves for sand without additives (stars) and for sand with 0.3 vol% of added gels: diaper gels Huggies (circles), GooN (triangles), Merries (squares), and agricultural gel Aquasorb (diamonds). The samples were preliminarily saturated with water. Full lines represent the fit of the experimental data using the Van Genuchten model [39]. Dotted lines are the same as in Figure 4a.

The water retention capacity of soil is usually characterized by the water retention curve (also known as pF curve), which describes a correlation between the water content in the soil W and the water pressure head at a given location in the soil [39] (Figure 4a). The higher the water content in the system under a given pressure, the better its ability to accumulate and retain water.

To vary the pressure during the experiments, equilibrium centrifugation was used [41]. Water retention curves obtained are shown in Figure 4b. Pure sand without additives was taken as a control. It is seen that the curves for all gel/sand mixtures are shifted to the right compared to pure sand, which indicates an increase in the water-retention capacity of the samples containing gels. Before centrifugation, at minimum external pressure, the water retention for pure sand is 22%. In the presence of gels, it increases to 77% (Merries), 81% (Aquasorb), 82% (GooN), and 89% (Huggies). Therefore, diaper gels provide the same increase in water retention as the agricultural gel. For all systems, increasing pressure reduces the water retention capacity W. The decrease for diaper gels is similar to that for the agricultural gel (Figure 4b). Thus, over the entire range of pressure examined, diaper gels do not appear to be inferior to agricultural gel in terms of their water retention capacity.

From the water retention curves (Figure 4a), several important hydrophysical characteristics can be derived [34,38,41,42,43]. First, this is the field water capacity (FWC), which represents the amount of water that is retained in the soil by adsorption and capillary forces after excess rain or irrigation water has been drained away [42]. Second, this is the wilting point (WP), which characterizes the moisture that is inaccessible to plants as it is firmly held at the surface of soil particles [42]. Third, this is the available water range (AWR), which represents the range of moisture that is available to plants. To estimate these characteristics, two straight lines corresponding to the following equations: pF = 2.17 + W/100 (line 1) and pF = 4.18 (line 2) are drawn (Figure 4a). The intersection of the water retention curve with line 1 gives the FWC value, whereas the intersection with line 2 yields the WP value [41,43]. The difference between FWC and WP values determines the range of moisture available to plants: AWR = FWC − WP. The values thus obtained are summarized in Table 3. One can see that, for diaper gels, the available water range for plants is almost the same as for the agricultural gel and much higher than for sand. Therefore, they are as effective as Aquasorb in providing water for plants.

**Figure 5:**
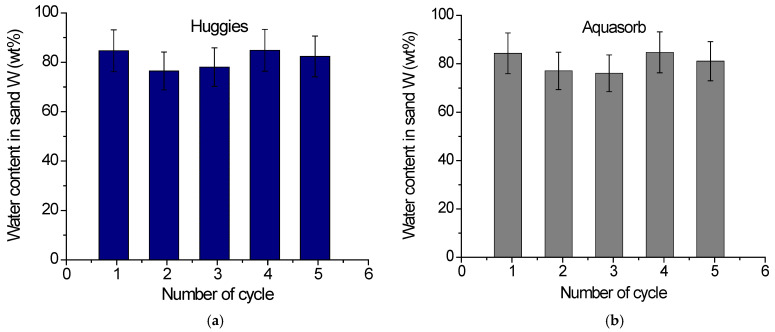

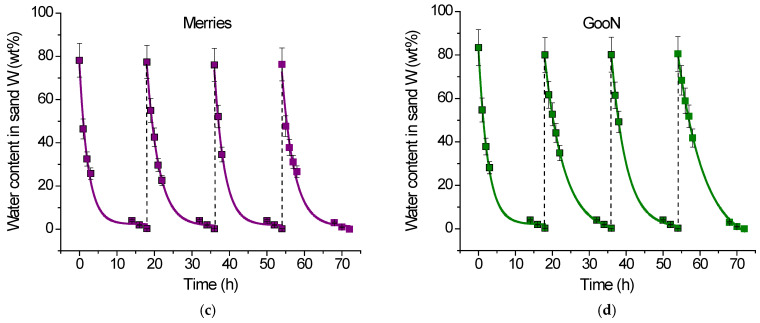
(**a**,**b**) The variation in the water content W in gel/sand mixtures containing 0.3 vol% of Huggies (**a**) and Aquasorb (**b**) gels at reswelling after losing the absorbed water due to centrifugation. (**c**,**d**) Kinetics of cyclic change in water content W in gel/sand mixtures containing 0.3 vol% of Merries (**c**) and GooN (**d**) gels upon drying at 30 °C and further reswelling upon immersion in water.

In soil, SAP gels undergo numerous swelling/drying cycles during their application. It was therefore important to study whether they can maintain their water-retention capacity under repeated swelling/drying cycles. To this end, two approaches were used. In the first approach, several water retention curves were measured consecutively for the same sample expelling the absorbed water at high pressure during centrifugation and then reswelling at ambient conditions. In the second approach, the swelling/drying cycles were repeated several times at constant pressure and temperature while monitoring the kinetics of the swelling and drying processes. Figure 5a,b illustrates the results of the first approach for two gels: diaper gel Huggies and agricultural gel Aquasorb. One can see that after five swelling/deswelling cycles the water content decreases by 2.7% for Huggies gel and by 3.8% for Aquasorb gel, which is within the margin of error. Figure 5c,d illustrates the results of the second approach for diaper gels Merries and GooN. One can see that, after four swelling/drying cycles, the water content in sand decreases very slightly: by 2.4% for Merries and by 3.3% for GooN, which also fall within the margin of error. These results indicate that good water-holding properties are maintained during several swelling/reswelling cycles for all the gels under study.

Note that, in this study, we chose sand as a model for soil because sandy soils have less available soil moisture than other types of soil (e.g., clay, loam) [44]. Therefore, the need for SAP for sandy soils is greater, and the impact of SAP on them is more significant, as has been demonstrated for various SAPs previously [33].

Thus, water-swelling and water-retention studies have shown that diaper gels significantly increase the water content in sand, and a large portion of this water is available to plants. These findings demonstrate a high potential for using diaper gels as water-retaining soil amendment.

### 2.3. Swelling Pressure

At swelling in sand pores, the swelling pressure of hydrogels plays an important role in counteracting the confining stress transmitted by sand particles. For this reason, the swelling pressure of diaper and agricultural gels was compared.

**Figure 6:**
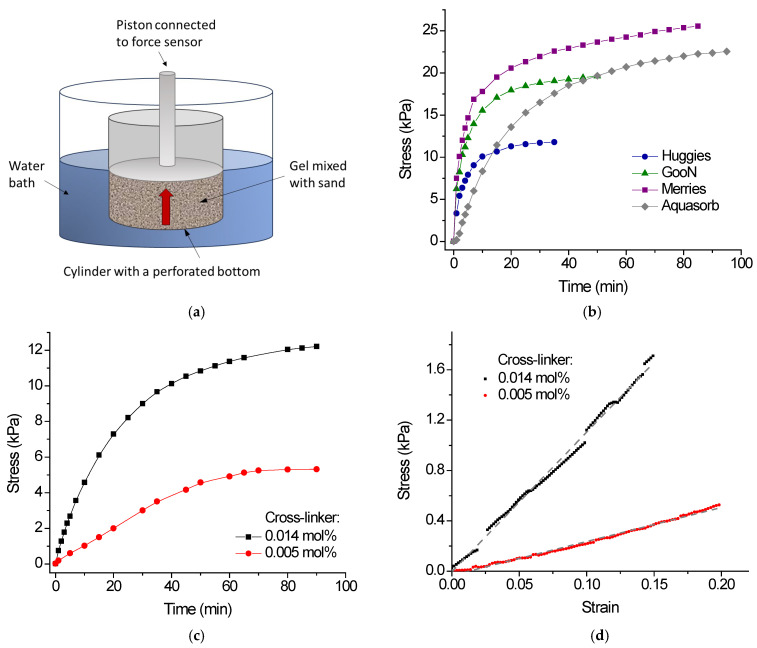
(**a**) Schematic representation of the setup for swelling pressure measurements. (**b**) Evolution of swelling pressure with time for 0.3 vol% dried diaper gels Huggies, GooN, Merries, and agricultural gel Aquasorb mixed with sand of a bulk density of 1.57 g/cm^3^. (**c**) Variation in swelling pressure over time for 0.3 vol% PAAM-SA gels with different crosslinking densities (0.005 mol% (red circles) and 0.014 mol% (black squares)) mixed with the same sand. (**d**) Stress–strain curve for compression of PAAM-SA gels with different crosslinking densities: 0.005 mol% (red circles) and 0.014 mol% (black squares). Dotted lines represent a linear approximation of the curves. The compression modulus E was determined from the slope of the curves.

Figure 6a shows a schematic representation of the setup for measuring swelling pressure [8,15,45]. The main component of this setup is a cylinder with a porous bottom, which allows for the exchange of water. Inside the cylinder, a sample of dry gel mixed with sand is placed on a filter paper. At the top of the sample, a piston with a measuring sensor is installed. The cylinder is placed in a reservoir filled with water, ensuring that the water level matches the height of the sample inside the cylinder. This is done to prevent the effect of water hydraulic pressure on the sensor readings. When the swelling occurred, the gel/sand sample pushed the force sensor upwards, and this force was recorded. This technique allowed us to monitor the evolution of the swelling pressure over time and to estimate the maximum swelling pressure reached (Figure 6b). The values of maximum swelling pressure are summarized in Table 2.

The swelling pressure of the diaper gels decreases in the order Merries > GooN > Huggies (Figure 6b), whereas, for free swelling, the trend is reversed: Huggies > GooN > Merries (Figure 3a). Considering that the content of charged groups in all three diaper samples is almost the same (Table 1), we can suggest that the higher degree of free swelling of the Huggies gel in water may be due to its lower cross-linking density [46]. However, lower cross-linking density provides weaker elasticity of gel, which cannot effectively compete with durable sand particles [15], and therefore exerts a lower swelling pressure. More cross-linked gels like Merries have a lower degree of free swelling in water but are mechanically stronger. This allows them to more effectively compete with sand particles, exerting higher swelling pressure. As a result, among diaper gels, the faster swelling and the larger swelling pressure are observed for Merries gel, which demonstrates lower free swelling. Thus, when gel particles are located in the pores between sand particles, swelling depends not only on the degree of charging and cross-linking density (as in the free swelling) but also on the balance between the mechanical strength of the swollen polymer and the resistance of the sand particles. Since the resistance of sand particles is the same for all samples, a higher swelling pressure may indicate a higher mechanical strength of the swollen polymer, which can be due to its higher cross-linking density.

To check the suggestion about the role of the cross-linking density in swelling pressure, two samples of gels differing in cross-linking density were synthesized by free-radical copolymerization of acrylamide and sodium acrylate. The gels were cut into small pieces so that, when dried, they were approximately the same size (0.40 mm) as other gel particles under study (Table 1). It was observed that the slightly cross-linked gel PAAm/SA-1 containing 0.005 mol% of cross-linker had a much higher degree of free swelling but a lower swelling pressure (Table 2) compared to the more strongly crosslinked gel PAAm/SA-2 with 0.014 mol% of cross-linker. Most obviously, this is due to the higher mechanical strength of the latter gel. Indeed, compression tests demonstrate (Figure 6d) that the elastic modulus E of PAAm/SA-2 gel (9.7 kPa) is much higher than that of PAAm/SA-1 gel (2.7 kPa). Thus, the higher the compression modulus of the gel, the greater the swelling pressure generated in a confined space within the sand. Indeed, when swelling inside the granular medium, the gel undergoes elastic deformation due to contact with solid particles [19]. Therefore, the swelling pressure should be proportional to compression modulus E. Note that this is the first paper that demonstrates the correlation between the swelling pressure and the compression modulus E of swollen gels. This correlation allows us to suggest that, among the three types of diaper gels under study, Merries gel exhibiting higher swelling pressure has a higher mechanical strength.

When the swelling pressure reached a constant value, the gel/sand samples were weighed in order to determine the degree of swelling in the sand pores (Table 2). It appeared that despite the different maximum swelling pressure exerted by diaper gel samples their degree of swelling in the sand pores is approximately the same. It may indicate that these gels are not strong enough to restructure and displace sand particles when they swell under a piston (Figure 6a).

When comparing swelling pressure curves for diaper and agricultural gels (Figure 6b), one can see that diaper gels swell faster and the maximum swelling pressure for some of them (Merries) is higher than for agricultural gel. The characteristic swelling time τ was determined from the exponential fit of the swelling pressure curve *S*(*t*) [47]:

(3) $$S\left(t\right)=S_{max}\left(1-{\mathrm{e}}^{-\frac{t}{\tau }}\right)$$

where $S_{max}$ is the maximum swelling pressure. For diaper gels, the swelling time τ is much shorter than for Aquasorb: 3.8 min for Huggies, 4.2 min for GooN, 4.9 min for Merries compared to 24.4 min for Aquasorb. This is an important advantage of diaper gels. Fast gel swelling is essential for agricultural applications, particularly in sandy soils. In such soil, water penetration occurs very quickly, resulting in a high infiltration rate [48]. If the water is not retained rapidly by the gel, most of it will be lost to the plants [8].

Thus, diaper gels are very promising for use in agriculture for water accumulation and retention to support plant growth.

### 2.4. Germination and Seedling Growth

Seed germination and seedling growth are essential processes in the development and establishment of vegetable plants. In this study, these processes were characterized by four parameters: total fresh weight of plants, shoot length, germination percentage and germination index. The germination percentage is the percentage of germinated seeds out of the total number of seeds used in an experiment [32]. Germination index takes into account both the number of germinated seeds and their root length, according to the following formula [49]:

(4) $$G_{index}=\;\frac{G}{G_{0}}\cdot \frac{L}{L_{0}}\cdot 100\%\;$$

where G and $G_{0}$ are the number of germinated seeds in the presence and absence of gel, respectively, L and $L_{0}$ are the root lengths in the presence and absence of gel, respectively.

Table 4 shows that both seed germination and seedling growth are improved by adding SAPs to the soil. The germination percentage as well as the total fresh weight of plants grown with all diaper gels are even higher than those grown with Aquasorb, while the shoots are longer with the latter. Overall, the effects of diaper gels on seed germination and seedling growth are comparable to those of agricultural gel. It is important to note that the germination index $G_{index}$ for the gels tested was well above 60%, which is the limit to consider the gels not phytotoxic [50]. Therefore, all studied gels do not show phytotoxicity and are safe for plants.

## 3. Conclusions

**Figure 7:**
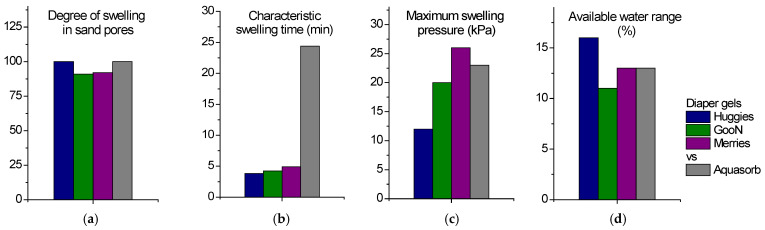
A schematic comparison of diaper SAP gels and agricultural gel Aquasorb mixed with sand (0.3 vol% of gel) in terms of degree of swelling (**a**), characteristic swelling time (**b**), maximum swelling pressure (**c**), and available water range (**d**).

The paper presents a comparative study of the water retention capacity of SAP gels removed from different types of diapers and the agricultural gel Aquasorb. The results are summarized in Figure 7. They show that diaper gels mixed with sand have the same ability to accumulate and retain water as agricultural gel, and similar swelling pressure. Even at low content such as 0.3 vol%, both diaper and agricultural gels significantly increase the available water range, which is responsible for plant germination and development. Therefore, diaper gels can be as effective as Aquasorb for soil moisture enhancement. In addition, diaper gels swell faster in sand pores compared to agricultural gel (Figure 7). This is an important benefit for sandy soils, which have a high infiltration rate and therefore require quick water retention by the gel. Also, the fact that diaper SAP gels have twice the charge density of agricultural gel makes them more suitable for fertilizer release applications, particularly for the release of cationic nutrient ions. Seed germination and seedling growth tests have demonstrated that the ability of diaper gels to enhance these processes is comparable to that of agricultural gel Aquasorb and that diaper gels are safe for plants.

Note that approximately 280,000 tons of SAP gels are used annually for the needs of agriculture; this accounts for about 12% of the total SAP production for diapers [3,4]. Therefore, if SAPs from diapers were reused, they could displace agricultural gels, eliminating the need for producing SAPs specifically for agriculture. This would conserve resources and reduce the overall amount of polymer waste.

Nowadays, the process of reusing diaper SAPs involves sterilization to eliminate pathogens, recovery of SAPs, and washing them with water [5,30,31,32]. The sterilization is performed by heating in an oven for 15 min at 125 °C [30,31,32], or by adding sodium hypochlorite as a disinfectant [5]. The recovery of SAP from the absorbent pads of diapers, which consist of cellulose pulp mixed with SAP, is carried out manually. This can be done by gently shaking the dried pad [30,32] or by adding calcium chloride to the wet pad and then stirring it with water [5]. In the latter case, it was estimated that about half of the SAP can be recovered from the cellulose pulp [5]. One can suggest the reuse of a whole absorbent pad of diapers, without SAP recovery. For example, such diaper pieces can be used in garden beds. After multiple uses, they can easily be removed from the soil and recycled.

At the same time, it should be acknowledged that the practical implementation of large-scale reuse of diaper SAPs may take a long time, given potential challenges related to the recovery process, costs, hygiene concerns, and regulatory barriers.

## 4. Materials and Methods

### 4.1. Materials

Three types of diapers were used: Huggies Dry Nights, size XXXL (Jaromer, Czech Republic), GooN Premium Soft, size L (Daio Paper Corporation, Fujimi, Japan), Merries Walker, size L (Kao Corporation, Tokyo, Japan). To recover the gel particles from the diaper, the absorbent pad was cut up and gently shaken to separate dried gel particles from the cellulose fibers. The results of the elemental analysis of the diaper gel samples are presented in Table 5. Nitrogen was not detected in any of the samples. Agricultural gel Aquasorb 3005 KM was purchased from SNF (Andrézieux, France). Medium-grained monomineralic quartz sand with a grain size of 1–1.6 mm (Polimerpro, Kirov, Russia) and a bulk density of 1.57 g/cm^3^ was used as received. The volume fraction of pores in the sand is equal to 0.42, taking into account that the density of quartz is 2.7 g/cm^3^.

Acrylamide (purity > 98%), sodium acrylate (purity > 99%), ammonium persulfate (purity > 98%), N,N,N′,N′-tetramethylethylenediamine (purity > 99%) from Sigma Aldrich (St. Louis, MO, USA) and N,N′-methylenebisacrylamide (purity > 99%) from Fluka (New York, NY, USA) were used as received. All solutions were prepared using distilled, deionized water purified by the Millipore Milli-Q system (Burlington, MA, USA).

### 4.2. Gel Synthesis

Hydrogels were synthesized by free-radical copolymerization of acrylamide and sodium acrylate in water, with N,N′-methylenebisacrylamide as a cross-linker and ammonium persulfate as an initiator [51]. After mixing all the components, a catalyst (N,N,N′,N′-tetramethylethylenediamine) was added, and the polymerization was carried out for 8 h at room temperature.

After polymerization, the gels were placed in a large volume of distilled water for few days to remove any unreacted chemicals. During this time, the outer solution was changed several times. For the synthesized gels, the sol fraction was less than 0.5%, as determined by weighing the dried samples.

The swollen gels were cut into approximately equal-sized cubes (with a 3 mm side for the slightly crosslinked gel PAAm/SA-1 and a 2 mm side for the more strongly crosslinked gel PAAm/SA-2). As a result, both synthesized gels in their dried state had the same size of 0.40 ± 0.04 mm, with a rather narrow size distribution.

### 4.3. Potentiometric Titration

Potentiometric titration was performed on pH meter Seven Multi Mettler Toledo (Schwerzenbach, Switzerland) in order to determine the content of charged groups in the gels. For the titration, 12 mg of finely crushed gel particles were dispersed in 9.9 mL of water, and 30 mg of NaCl was added to maintain the ionic strength of the dispersion. Then, the pH was adjusted to 2.2–2.4 by adding 0.1 mL of 1 M HCl in order to protonate the carboxylic groups. After 1 h, the dispersion was titrated with 0.1 M NaOH [34]. The concentration of carboxylic groups (in mmoles per 1 g of dried gel) C was determined as [52]:

(5) $$C=\frac{(V_{2}-V_{1})\cdot C_{NaOH}}{m}$$

where $V_{2}$—volume of titrant at the second inflection point corresponding to the titration of gel carboxylic groups, $V_{1}$—volume of titrant at the first inflection point corresponding to the titration of HCl excess, $C_{NaOH}$—the concentration of titrant, and m—mass of dried gel.

### 4.4. Free Swelling

For free swelling studies, ca. 20 mg of dried gel particles were immersed in 60 mL of distilled water. The degree of swelling of water-swollen gel particles was determined gravimetrically using Equation (1). All experiments were performed in triplicate.

### 4.5. Water Retention

For water retention experiments, the 0.3 vol% mixture of dried gel particles with the sand containing 0.02 g of dry gel and 10.46 g of sand was placed in nylon filter bags and immersed in water until an equilibrium degree of swelling was reached. To create external pressure, the equilibrium centrifugation was used [41]. The samples were centrifuged sequentially at different speeds of *n* (300, 400, 600, 800, 1000, 1300, 2000, 3000, 5000 rpm). The pressure of water during centrifugation was estimated using the following formula [41,43]:

(6) $$\left|P\right|=-(0.011\cdot n^{2}r\cdot \cos\alpha +g\sin\alpha )\cdot h$$

where *n* is the number of centrifuge rotations per minute, *r* is the distance from the rotation axis to the center of mass of the sample, *h* is the height of the sample, and α is the angle between the horizontal axis and the central axis of symmetry of the sample. $\left|P\right|$ is the absolute value of the soil water pressure, which has a negative sign by definition [43]. The experiments were performed with a laboratory centrifuge, Thermo Scientific SL 16 (Langenselbold, Germany). After each centrifugation, the weight of the remaining water W was measured. The pF(W) curves obtained were approximated by the van Genuchten model, which describes their S-shaped forms with a minimum number of parameters [39]. Three independent samples were tested for each condition. The error bars represent the standard errors of the mean.

### 4.6. Swelling Pressure and Swelling in Sand Pores

Swelling pressure experiments were performed on Lloyd LS5 tensile machine (Ametek, Berwyn, PA, USA) using a setup presented in Figure 6a and a force sensor with an accuracy of ±0.1%. To conduct these experiments, 0.111 g of dry gel mixed with 57.9 g of sand (0.3 vol% of gel) was placed into metal cylinder with a perforated bottom covered with filter paper (Figure 6a). The height of sample layer was 1.5 cm. Next, a piston attached to the force sensor was lowered until it came into contact with the mixture. The piston’s diameter matched the inner diameter of the cylinder. After that, water was added to the outer bath to a level equal to the height of the mixture, and the pressure on the plunger exerted by swelling mixture was measured over time.

At the end of the experiment, the swollen gel/sand mixture was weighed, and the degree of swelling of gel particles in sand pores was determined using Equation (2). All experiments were performed in triplicate.

For the study of the swelling/drying kinetics, we used nylon filter bags to place 0.02 g of dried gel thoroughly mixed with 10.46 g of sand (0.3 vol% of gel). The bags were immersed in water until equilibrium swelling was reached. Then, they were dried in an oven at 30 °C to a constant weight, while the kinetics of drying were investigated. The swelling/drying cycles were repeated four times.

### 4.7. Mechanical Characterization

Uniaxial compression measurements were performed on Lloyd LS5 tensile machine (Ametek, Berwyn, PA, USA) equipped with a 100 N load cell at room temperature. The equilibrium swollen gels in the form of cylindrical discs (diameter 12–25 mm, height 10–20 mm) were used. Each measurement was repeated at least 3 times to obtain the stress–strain curve within the range of 0–20% strain in the elastic region of deformation. The compression modulus was calculated using the linear interval of the stress–strain curves [53].

### 4.8. Optical Microscopy

Optical microscopy measurements were carried out using a light microscope Nikon Eclipse LV100 POL (Tokyo, Japan) with a CFI TU Plan Fluor Epi objective lens (p 5×), at room temperature.

### 4.9. Germination and Seedling Growth

For seed germination and seedling growth experiments, seeds of Sarepta sideral mustard *Brassica juncea* L. (Gavrish, Moscow, Russia) were chosen. The SAP gels were mixed at a rate of 0.3 vol% into dried soil Terra forte (91% turf, 5% sand, 4% perlite) provided by Nevatorf (Fornosovo, Leningrad region, Russia). Then, 250 mL of gel/soil mixtures were filled in dishes. The soil without gel was used as a control. Mustard seeds were evenly distributed in the soil, and 100 mL of water was added. Dishes were placed in a dark room at 25 °C under plastic wrap for approximately 24 h before seed germination. After seed germination, the film was removed. No water was applied except for the initial saturation of the dishes. After 7 days of light exposure, the average length of the plant shoots was about 5 cm. For each sample, we determined the number of germinated seeds, the length of shoots and roots, and the total fresh weight of the sprouts. Then the germination percentage was calculated from the ratio of the number of germinated seeds to the total number of seeds [32], and the germination index was calculated using Equation (4).

## Figures and Tables

**Table 1:** Characteristics of commercial gel samples.

Gel Sample	Application	Size of Dry Gel Particles, mm		Content of Charged COO^-^ Groups, mmol/g
		Small Size Fraction	Large Size Fraction	
Huggies	diapers	0.2–0.25	-	3.6
GooN	diapers	0.2–0.25	0.55–0.6	3.7
Merries	diapers	-	0.4	3.5
Aquasorb	agriculture	0.15	0.55	1.8

**Table 2:** The equilibrium degree of swelling and the maximum swelling pressure of hydrogels in sand pores, as well as the equilibrium degree of free swelling of hydrogels in water.

Gel Sample	Equilibrium Degree of Swelling		Maximum Swelling Pressure *, kPa
	Swelling in Sand Pores *	Free Swelling	
Huggies	100 ± 10	540 ± 25	12 ± 1
GooN	91 ± 9	450 ± 20	20 ± 2
Merries	92 ± 9	380 ± 20	26 ± 2
Aquasorb	100 ± 10	310 ± 15	23 ± 2
PAAm/SA-1	90 ± 9	420 ± 20	5 ± 1
PAAm/SA-2	96 ± 9	140 ± 10	12 ± 1

* 0.3 vol% of gels in sand.

**Table 3:** Hydrophysical characteristics of sand loaded with 0.3 vol% of different dried gels.

Gel Sample	Field Water Capacity FWC, %	Wilting Point WP, %	Available Water Range AWR, %
Huggies	37	21	16 ± 2
GooN	33	22	11 ± 2
Merries	28	15	13 ± 2
Aquasorb	35	22	13 ± 2
No gel *	4.1	0.4	3.7 ± 0.5

* Control sample (sand without gel).

**Table 4:** Growth parameters of mustard plant grown on soil with 0.3 vol% of different dried SAP gels.

Gel Sample	Total Fresh Weight, g	Shoot Length, mm	Germination Percentage, %	Germination Index, %
Huggies	2.1 ± 0.2	42 ± 2	62 ± 2	137 ± 10
GooN	2.5 ± 0.2	52 ± 3	58 ± 2	158 ± 12
Merries	2.3 ± 0.2	44 ± 2	72 ± 3	163 ± 12
Aquasorb	2.0 ± 0.2	59 ± 3	54 ± 2	171 ± 14
No gel *	1.3 ± 0.1	37 ± 2	50 ± 2	100

* Control sample (sand without gel).

**Table 5:** Elemental analysis for diaper gels.

Gel Sample	C, %	H, %	N, %
Huggies	37.67	4.42	0
GooN	38.34	4.55	0
Merries	33.67	5.25	0

## Data Availability

The data presented in this study are openly available in article.
